# Mapping Public Engagement with Research in a UK University

**DOI:** 10.1371/journal.pone.0121874

**Published:** 2015-04-02

**Authors:** Ann Grand, Gareth Davies, Richard Holliman, Anne Adams

**Affiliations:** 1 Institute of Educational Technology, The Open University, Walton Hall, Milton Keynes, MK7 6AA, United Kingdom; 2 Faculty of Science, The Open University, Walton Hall, Milton Keynes, MK7 6AA, United Kingdom; Institute of Infectious Disease and Molecular Medicine, SOUTH AFRICA

## Abstract

Notwithstanding that ‘public engagement’ is conceptualised differently internationally and in different academic disciplines, higher education institutions largely accept the importance of public engagement with research. However, there is limited evidence on how researchers conceptualise engagement, their views on what constitutes engagement and the communities they would (or would not) like to engage with. This paper presents the results of a survey of researchers in the Open University that sought to gather data to fill these gaps. This research was part of an action research project designed to embed engagement in the routine practices of researchers at all levels. The findings indicate that researchers have a relatively narrow view of public engagement with research and the communities with which they interact. It also identified that very few strategically evaluate their public engagement activities. We conclude by discussing some of the interventions we have introduced with the aim of broadening and deepening future researcher engagement.

## Introduction

### The historical context

The route to engaged research undoubtedly varies according to academic discipline and the journey is characterised by the relationship between knowledge, its producers and those affected by the research. Here, we briefly explore 70 years of development in relation to the sciences and their publics as a way of illustrating the significance of the research we have conducted.

After World War II ended, progress towards the ‘endless frontier’ of research—and especially scientific research—was seen as the means by which nations would ensure their peoples’ future health, prosperity and security.[1] In return for these goods, the people’s role was to sanction research funding; effectively, to endorse researchers’ licence to practise.[2] Further discussion of the reasoning behind the development of a relationship between professional scientists and the public centred on the idea that part of the duty of being a scientific researcher was a responsibility to improve understanding, communicate the technological, humanitarian and economic benefits of science and contribute to better-quality public and private decision-making.[3] In this formulation, the relationship between scientists and the public was largely conceptualised as educative: the scientists’ main purpose for communicating was to school a scientifically illiterate public.[4]

Despite the obligations placed on scientists and the perceived economic value of increasing public understanding, by the mid-twentieth century the scientific establishment was becoming concerned by scientists’:

‘mistrust, lack of understanding and often unwillingness and inability to communicate adequately’ and their tendency to ‘retreat into their shells, frowning on those who ventured onto the public stage’.[3] (para 6.1)

One of the significant implications of this judgement was that without scientists’ active participation in society, funding for scientific research could be politically vulnerable.[5]

Alongside these economic considerations grew concerns that the role of scientists as specialised keepers of knowledge, healers of ignorance and assuagers of deficits was neglecting the considerable, if informal and localised, understanding and expertise possessed by members of the public.[6] Survey evidence indicated people were willing to learn more about science and to discuss its ethical and social implications,[7] often because they considered scientists would benefit from listening more ‘to what ordinary people think’.[8] (p.52) This implied that scientific researchers needed to do more than simply tell people what they were doing; they also needed to listen to people and respond, even if they considered their antagonism, fears or hopes to be ill-founded.[9] Further evidence, predominantly from qualitative accounts, showed that researchers could gain from drawing on the knowledge and experiences of other expert stakeholders and members of the public, in effect valuing different kinds of expertise as additional resources to inform the overall research endeavour; the collaborative sum would be greater than researchers working only with other academic researchers.[10]

Over time, the rhetoric shifted from understanding to engagement. This movement is encapsulated in the definition developed by the UK’s National Co-ordinating Centre for Public Engagement (NCCPE), which defines engagement as ‘a two-way process, involving interacting and listening, with the goal of generating mutual benefit’.[11]

### The complexities of public engagement with research

Public engagement with research takes multifarious and diverse forms. The New Economics Foundation’s *Participation Works*![12] detailed 21 participatory mechanisms; Wilsdon and Willis[13] recorded activities including deliberative polling, focus groups, citizens’ juries, consensus conferences, stakeholder dialogues, Internet dialogues and deliberative mapping; Rowe and Frewer[14] identified around a hundred participatory activities; Mesure[15] approximately 1500 initiatives; DIUS [16] noted growing numbers of science and discovery centres, museums, cafes scientifiques and festivals. Bauer and Jensen[17] extended engagement to include public lecturing, giving interviews, writing popular books or articles and collaborating with non-governmental organisations and Davies added ‘volunteering activities, participatory social research, [and] even informal conversations about research outside the university setting, as well as more familiar activities such as giving talks to school groups or holding university open days’.[18] (p.726)

These many activities suggest that at least to some degree researchers and citizens are willing to engage through dialogue and other forms of participation, but nevertheless, some important questions remain. First come the practical challenges: who should (and should not) engage, when and how often, how should the engagement be organised, for what purpose(s), how will success or failure be measured and how should the engagement (and contributions of the participants) be funded?

Second is the disruptive challenge of acknowledging and accrediting expertise within existing systems of academic validation, for example peer-reviewed publication. For some, accepting the idea that ‘publics, not only the scientists or experts, can make useful and valuable contributions to discussions and decisions about science and technology’,[19] (p.13) challenges hard-won expertise and raises questions about how researchers can ensure that the contributions of all participants in a research project are recognised in ways that are meaningful and useful.

Third, how might researchers’ professional identities be affected by adopting a more open and distributed approach to research? Researchers may well have spent many years developing their knowledge, skills and craft; being seen by peers and others as an expert is an important part of professional identity:

Involving the public inevitably means researchers have to give up some of their power [and] although many researchers have recognised that this shift is essential for projects to become genuinely collaborative, no one has reported finding it easy. [20] (p.66)

A fourth challenge relates to the question of who owns research; for researchers, their professional value may depend on their being in control of ideas, data and intellectual property. Many agencies have an interest in the value of research and its outputs: researchers themselves, their institutions, funders, industry, etc.; extending ownership by inviting wider engagement may be to the advantage of the research but could be seen as diluting the return on investment. Fifth, despite descriptions of dialogue as a two-way communication, the extent of genuine reciprocity and dialogue in public engagement activities is debated.[21] In practice, tension persists between assumptions about deference, expertise and scientific privilege[22]—the language of dialogue, debate and lay agency—and evidence for the appropriateness of, and public preference for, listening rather than talking in certain circumstances.[23, 24] As Holliman et al., argued:

It is possible to imagine ‘deficit desire’—audiences who actively seek the linear lecture from a trustworthy expert with little expectation or interest to challenge the scientific perspective presented to them—as well as ‘dialogue fatigue’—those who actively avoid forms of engaged democratic citizenship in relation to science. And they could be the same person. [25] (p.276)

In other words, genuine engagement requires not only that publics are given input into the topics of research but also that the methodologies for engaging are considered collaboratively. This is not to say that engagement is synonymous with democracy, at least not straightforwardly. Engaged research requires different forms of expertise, whose relevance will wax and wane depending on the research and on the point in the research cycle where engagement happens.

A final challenge for researchers and institutions in the UK is that the concept of engagement has the potential to become entwined with the concept of measuring the impact of research. In 2014, the Research Excellence Framework (REF) reported on the quality of research in UK higher education institutions.[26] Although public engagement with research is often conceived as the reification of a co-operative relationship among institutes of higher education (HEIs), the communities within which they sit and stakeholders with an interest in the outcomes of the research, for example through HEIs sustaining local economies,[27] it has, to varying degrees, also been identified both as a mechanism for providing evidence of the impact of research, and as itself being a form of impact.[28] In the REF, the efficacy of the work of academics within UK universities was assessed to determine the reach and significance of the engaged research’s public value and impact. As we noted above, in the past, public engagement with research has served multiple purposes, for example as a vehicle for increasing openness and transparency, as a means for maintaining support for the use of public money for research,[2] enhancing citizens’ discussion of scientific issues,[7,8] incorporating public concerns and skills in research[10] or encouraging more students to take up specific subjects such as the sciences.[29] However, the potential long-term effect of the REF could instead be to transform public engagement into the ‘engine powering the conceptual, critical and methodological framings and motivations of impact’.[27] (p.120) Our counter to this challenge is to foster and encourage progressive approaches that ultimately improve the quality of research in ways that are meaningful to all participants.

### Context of this research

Many interventions across the UK higher education sector have been designed to address the relationship between universities and other sectors of society. From 2008–2011, RCUK, the UK Higher Education Funding Councils, and the Wellcome Trust funded the Beacons for Public Engagement initiative. Six ‘beacons’, university-based collaborative centres, were established to support, recognise, reward and build capacity for public engagement. The NCCPE, also established under the Beacons for Public Engagement initiative, was set up to co-ordinate and share learning among the Beacons and across UK higher education institutions and research institutes.

In 2011, RCUK issued a more narrowly-focussed call to fund eight ‘Public Engagement with Research’ (PER) Catalysts over three years (completing March 2015). The PER Catalyst initiative invited universities to draw on and develop the learning from the Beacons programme, with the overarching aim of embedding a culture within universities where public engagement with research is strategically planned, systematically assessed, valued and recognised.

The Open University (OU) received funding under the PER Catalyst for action research to develop and implement strategies that promote structured and equitable mechanisms for effective and sustainable engagement with a range of publics, stakeholders and user communities. To achieve this, the project team proposed a programme of organisational change in which OU researchers were given opportunities to be involved and engaged as co-researchers. This programme of organisational change is informed by an action research approach,[30] initially involving rigorous research-based inquiry into how the university currently functions with respect to public engagement with research. Findings from this initial diagnostic exploration are the focus of this paper.

In conceptualising the plans for our project, ‘An open research university’, we found limited data on researchers’ conceptualisation of engagement, what activities they consider constitute engagement, what communities they believe they are engaging with and what communities they would (or would not) like to engage with.[31, 32] How do researchers’ views on engagement relate to the operation of public engagement with research and research practice within the university?

We have used the findings from this study, in combination with other data, to map researcher practices across the OU, investigating the processes of innovation that have led to the introduction of public engagement with research. We have collated existing resources and documented current practices to support researchers in several academic domains. This helped us to identify areas where additional interventions that have an impact on institutional systems and processes could be considered; we describe some in our Conclusions.

## Method

Our findings are derived from OU researchers’ responses to the 2013 Vitae Careers in Research (CROS) and the Principal Investigators and Research Leaders (PIRLS) online surveys. Vitae (www.vitae.ac.uk) is a not-for-profit organisation supporting the professional and career development of post-graduate researchers and research staff in UK higher education institutions and research institutes. It is funded by Research Councils UK, UK HE funding bodies and institutional subscription. CROS and PIRLS are biennial comparative surveys run by a group of UK universities to form a ‘benchmarking club’.[33] Each survey has a set of common core questions that is asked in all participating universities; the CROS question set is slightly different to that of PIRLS. In addition, universities have the option to add questions that are asked only within their own institution and the results of which are only available to that university. The OU’s CROS and PIRLS surveys were administered via the Open University’s staff networks. The surveys were run in May 2013 and were open for two weeks.

### Ethics statement

The research protocol was approved by the Open University’s Human Research Ethics Committee and the surveys were hosted on the Bristol Online Surveys platform, which provides a secure environment for the administration and analysis of online surveys.[34, 35] Neither the CROS nor PIRLS surveys asked respondents to give personal information such as names or contact details, although they were asked for data such as length of service and academic discipline. The institution-specific questions additionally stipulated that respondents should not include details that could inadvertently identify them, such as URLs or the names and locations of activities. All the institution-specific questions were free-text responses.

Participants were deemed to have consented when they submitted the questionnaire. The invitation to respond to the surveys included the following information:

“Your responses will be anonymous and you will not be identified or identifiable. You can complete [the survey] if [information identifying which survey (CROS/PIRLS) was appropriate]. It should only take about 20 minutes to complete; the information you provide will bring long-lasting benefit to you and your peers.

Participation in the survey is entirely voluntary and you can discontinue at any point up until the questionnaire is submitted. This is because no names are taken, so we cannot distinguish between individual participants’ data. All data is confidential and will be stored securely. By submitting or returning the questionnaire, you consent to take part in the survey and for your data to be included.

If you agree to take part in this survey, please click on the link below.”

### CROS survey

The CROS survey had 27 core questions, of which some were in several parts. A number of these were related to public engagement and the impact of research, including:

Q7f: To what extent do you agree that your institution both recognises and values the contributions that you make to public engagement with research?
Q12: How would you rate your knowledge and understanding of the following UK initiatives relevant to research staff?
  - Concordat for Engaging the Public with Research
  - RCUK’s Pathways to Impact
Q19: In which areas have you undertaken, or would you like to undertake, training and other continuing professional development activities?
  - Public Engagement with Research
Q25: Which of the following have you done or would like to do as part of your current role?
  - Engage with policy-makers and end users
  - Participate in public engagement activities

### PIRLS survey

The PIRLS survey had 19 core questions, of which some were in several parts. As with CROS, some were related to public engagement and research impact:

Q8a: My institution recognises and values the contribution I make to:
  - Demonstrating the impact of research
  - Public engagement and outreach activities
Q8b: I think this activity is very important in being a successful PI/research leader:
  - Demonstrating the impact of research
  - Public engagement and outreach activities

### Institution-specific questions

Professional development is an integral aspect of the action phases of our PER Catalyst project. In part to inform the requirements of future training needs, we consulted the OU’s Research Career Development Team at an early stage in the project to develop four institution-specific questions that were added to both the CROS and PIRLS surveys. These questions explored researchers’ understandings of publics and public engagement with research:

The Open University is working to create a culture in which public engagement with research is embedded within strategic planning for research and the operational practices of researchers at all levels. In fewer than 150 words, how would you define ‘public engagement with research’?
Please describe, in general terms, a successful activity involving public engagement with research in which you have participated and the criteria you used for judging the success of the activity.
  - (a) Could you describe what non-academic communities or people you consider have connections with your research, either currently or in the past?
  - (b) Could you describe what types of publics would you like to engage with your research?
  - (c) Are there publics that you would not choose to engage with? If so, please describe why.
Please describe your three most important reasons for engaging with the non-academic communities or people that you listed in question 3a.

### Coding and analysis

Responses for the selected questions from the core sets were downloaded and imported into SPSS21, which was used to store and analyse the data. The majority of responses were categorical variables that could be summarised as univariate measures. Respondents were free to choose whether or not to answer any particular question.

The free-text responses were analysed as qualitative data. We acknowledge that by using free-response questions to collect qualitative data, we did not have the opportunity to probe respondents to extract deeper meaning and validate responses using their own concepts, frames of reference and vocabulary. Using web-based surveys offers the opportunity to ask questions of a larger number of respondents than can feasibly be managed by interview. However, using surveys means we lose contextual information, such as the age and sex of respondents and the social context in which they are completing the survey.[36, 37] In the project as a whole, we used a mixed methods approach, triangulating quantitative with qualitative approaches (a series of semi-structured interviews) to complement each other and support validation.[32] However, in this paper we are not reporting on the interview data.

The analysis began with the inductive construction of coding categories. The coding categories were emergent; that is, they were formulated during analysis and grounded in the data, rather than being defined beforehand. Although we acknowledge that no one can approach analysis with a completely open mind, a data-grounded approach to analysis allows the researcher to start with fewer preconceptions.[38] To minimise bias that might result from an individual’s personal conceits and existing notions, two researchers independently printed, read and scrutinised the free-text responses for key concepts, actions, relationships and meanings. The resulting codes were applied to the responses, then refined and edited to remove overlaps, confusions and repetitions. This process was repeated until no further changes were made to the codes. Finally, the two researchers agreed a set of codes and definitions (see Tables 1, 2, 3 and 4).

**Table 1:** Researchers’ definitions of ‘public engagement with research’.

**Code**	**Description**	**%**	**n**
Dissemination	*Dissemination/communication/presentation*: Through appropriate language and a variety of one-way communications, ensuring wider (e.g. non-academic) audiences can receive information about the process of research and research findings; outreach; talks/lectures; explaining, clarifying, translating, simplifying or educating.	32	54
Collaboration	*Collaboration/participation/consultation*: Involving people in research from the inception of projects; affording people the opportunity to understand, participate and shape research priorities and the design of projects; consulting groups that want to do something with the research.	11	19
Dialogue	*Dialogue/exchange of ideas*: Engaging in dialogue or exchanging ideas with a diverse range of audiences/user groups/specialist researchers/interested parties/publics; enhancing mutual benefit by listening/participating in ways that help shape/reshape the social demand and understanding of research; influencing policy.	8	14
Useful	*Demonstrating the usefulness/benefits of research*: Demonstrating the importance of research; enhancing people’s understanding of how research can affect their community and improve their lives (e.g. offering economic benefits); demonstrate economic value of research.	7	13
Functional	*Functional/strategic/occupational*: Sustaining resources and concrete targets in research projects; training researchers in engagement; offering media support; meeting institutional targets for public engagement; a defined part of the job role.	4	6
Non-participation	Antithetical/negative/dismissive views about public engagement with research.	1	2
Don’t know	Responses given as ‘don’t know’ or similar.	2	4
Unclassifiable	Responses that did not include a definition.	8	13
No answer	Respondents left the question blank.	27	46
Total		100	171

**Table 2:** Researchers’ descriptions of public engagement with research activities.

**Code**	**Description**	**%**	**n**
Presenting	Presentations/talks/lectures e.g. at festivals, fairs, exhibitions, roadshows, public meetings; radio/television programmes; to national and international audiences, special-interest groups, charitable and volunteer organisations.	19	33
Partnerships	Co-production of research with diverse groups; working with practitioners.	14	23
None	Respondents said they did not carry out any public engagement activities.	8	13
Activities	Workshops with non-academic groups; university events open to the public, practitioners, policy-makers, other researchers (e.g. seminars, open days).	7	12
Schools	Activities involving school students; outreach activities; talks/lectures in schools.	7	12
Digital	Writing research blogs; other social media activities; forums; citizen inquiry projects.	4	6
Writing	Writing for books, newspapers, magazines, policy documents.	2	4
Not possible	Unable to describe, as this would identify the researcher.	1	2
Unclassifiable	Responses that did not include a description of an activity	6	11
No answer	Respondents left this question blank.	32	55
Total		100	171

**Table 3:** Researchers’ reasons for undertaking public engagement with research.

**Code**	**Description**	**%**	**n**
Influence	To influence policy/policy-makers/decision-making; influence practice; create practical outcomes.	15.0	37
Improve research	Engaging with non-academic communities to develop a better understanding of research problems; increase relevance; keep real-world perspective.	11.8	29
Communication	Dissemination and communication about research; raise profile of research/researchers; raise awareness about research.	9.8	24
Funding	To increase access to funders.	7.7	19
Education	For educational purposes; increase understanding.	7.3	18
Enthuse	To enthuse specified groups (e.g. girls) to encourage take-up of specific subjects (e.g. science).	7.3	18
Access to people	Access to people interested/involved in research, research participants, target audiences, stakeholders; to break barriers between researchers and public.	6.9	17
Dialogue	Promote dialogue; share ideas/knowledge; share findings.	6.9	17
Obligation	Research is publicly-funded; should be publicly-disseminated/ communicated.	5.7	14
Show impact	Maximise socio-economic impact; maximise cultural/social impact; evidence for future funding.	4.9	12
Enjoyment	Enjoyment of public engagement with research activities.	4.5	11
Accountability	Demonstrate value; increase accountability and transparency; show benefits of research.	4.1	10
Collaboration	Collaboration with public/interest/community groups.	3.7	9
Inclusion	Ensuring knowledge is produced in a way that includes range of perspectives.	2.4	6
Requirement	Part of the job.	1.2	3
Skills development	Develop researchers’ communication skills.	0.8	2
Total		100	246
No answer	Respondents left this question blank		53

**Table 4:** Researchers’ criteria for judging success of public engagement with research activities.

**Code**	**Description**	**%**	**n**
None	Respondents gave responses such as ‘none’ or ‘no criteria’	42	39
Follow-up	Post-event feedback from participants; emails or other communications; interest in OU courses; interest from media.	19	18
Reception	Behaviour at the event: levels of participation; questions asked; informal feedback; evidence of enthusiasm and excitement.	16	15
Numbers	Numbers of participants/people reached.	6	6
Evaluation	Formal or semi-formal evaluation of the event.	5	5
Influence	Evidence of influence on policy or practice.	5	5
Unclassifiable	Responses that did not include a description of criteria	3	3
Visibility	Enhanced visibility in existing networks; invitations to give further talks/conference presentations; establishment of new research networks.	2	2
Total		100	93
No answer	Respondents left this question blank		92

## Findings: Exploring the Landscape of Public Engagement with Research

In this section, we discuss how researchers define public engagement with research, what activities they consider to be engagement and the communities with which they do (or do not) choose to engage. We also discuss some related data on researchers’ experiences of training for, and participation in, public engagement with research.

### Response rates

For both surveys, the response rate of OU researchers was broadly in line with the response rate of the national survey. Sixty-eight UK universities took part in the CROS survey. A total of 8216 complete, non-duplicate responses were received, an overall response rate of approximately 26% of potential respondents.[34] The Open University CROS survey received 57 responses (34% response rate). Forty-nine UK universities took part in the PIRLS survey. A total of 4837 complete responses were received, representing a response rate of 28%.[35] The Open University PIRLS survey received 114 responses (22% response rate).

Unless specifically stated, our findings are based on researchers’ responses (57 + 114 = 171 in total).

### Defining and practising public engagement with research

We begin with the premise that language and practice are inextricably enmeshed. Therefore, in this section, we will compare researchers’ personal definitions of public engagement with research (see Table 1) with the types of public engagement activity they describe they undertake (see Table 2). Answers to the institution-specific questions showed that researchers vary in their conceptualisation of public engagement with research (see Table 1).

Most respondents (Table 1: 73%, n = 125) offered some kind of definition or description of public engagement with research, ranging from the personal (‘I enjoy giving public lectures’) to the utilitarian (‘I’m paid to do it’), to the philosophical (‘involving the public, as in Habermas’s conception of the public sphere’). A very small number (1%, n = 2) described engagement negatively, for example ‘a distraction from core business’. Thirty-nine per cent (n = 63) did not reply, did not provide a definition or said that they ‘didn’t know’. This latter finding suggests some researchers remain unaware of the recent shift in official rhetoric about public engagement with research.

#### Dissemination and communication

The most common definitions of ‘public engagement with research’ focussed on the dissemination, communication or presentation of research. Thirty-two per cent (Table 1: 32% n = 54) of researchers offered definitions that fitted within ‘dissemination’, for example:

*‘*The dissemination of both the goals and results of research to wider audiences, including non-academic’

‘Disseminating our research results to public and other institutions in a clear and understandable way *so that they can be used to help them achieve their aims*’ (emphasis added)

‘Dissemination of ideas and practical potential of research, and transference of these ideas into common sense understanding of the world by the public’

Dissemination is clearly a central code in this analysis. We interpret this to mean the respondents are conceptualising engagement in a mode that Irwin described as first-order: deficit-model, one-way, top-down communication.[10] Ideas are ‘transferred’ into public understanding, information is ‘conveyed’ and talks are ‘aimed’. As Davies argued of scientists, some persist in perceiving engagement as difficult, dangerous and framed within an over-arching context of one-way transfer.[39] On the other hand, characterising linear one-way communication as somehow inferior to dialogue ignores people’s desire for information and the enablement that can arise from understanding.[23, 25] There is also evidence of some conflation of terminology, which is also reflected in other studies.[31, 32, 40] Several responses included mixed terminology (see, for example the emphasis above), which could indicate the incorporation of dialogic methods within wider dissemination strategies, indicating the dominant dissemination view of engagement is reinforced by researchers’ descriptions of engagement activities in which they had been involved (see Table 2).

Approximately half (Table 2: 53%, n = 90) of the researchers were able to describe an activity they considered to be engagement. Although only 8% (n = 13) of researchers said they didn’t do any public engagement activities, a further 32% (n = 55) left the response blank, which may or may not indicate non-participation.

Those who did respond described a variety of activities, by no means all of which could be said to be framed by the NCCPE’s [11] vision of dialogue and mutuality. For example, 19% (n = 33) of researchers described researcher-led dissemination activities:

‘We took part in an RCUK event to showcase our work to the public’

‘Conveying to non-expert colleagues and to the wider public the content, scope and significance of my research’

‘Informing and explaining to the public about developments, potential developments and applications in research’

This seems to demonstrate that the language and rhetoric of engagement is being operationalised in specific and quite conservative ways; researchers ‘have begun to talk the talk of engagement, but have not started to walk the walk’. [41] (p.61) Our challenge, as an action research project, is to identify where this is so, and to invite researchers to explore more imaginative and relevant mechanisms to engage a wider set of stakeholders.

#### Partnership, dialogue and collaboration

Partnership, dialogue and collaboration are mentioned in both researchers’ definitions of public engagement with research (Table 1) and their descriptions of activities (Table 2). For example, 11% (n = 19) of researchers’ definitions (Table 1) could be coded as the ‘collaboration’ of different communities—researchers, non-academics, members of the public, stakeholders, policy-makers, etc.—in the process of research, for example:

‘Involving the public from the outset in the design, consultation, evaluation and distribution of a research project’

‘Collaborating with key stakeholders in the planning, execution and dissemination of research, ensuring that findings [are] presented in way relevant and accessible to key audiences’

‘Collaborating with 3rd sector, industry, media, government sectors to enable knowledge and skills exchange’

Collaboration was sometimes linked with dialogue; a small group of researchers (Table 1; 8%, n = 14) employed ‘dialogue’ or its equivalent in their definitions:

‘A process of dialogue and shared learning between researchers and others, those being researched as well as wider audiences and communities. In some contexts the co-production of research may be a useful model’

‘Engaging in and being open to dialogue with non-academic publics, not solely by giving talks but by listening/participating as well’

These descriptions were also reflected in the description of activities. The next most common description of engagement activities (Table 2: 14%, n = 23) was of ‘partnerships’ involving working with practitioners and co-production of research with diverse groups:

‘Public events that helped to shape/reshape the trajectory of a research project at its outset’

‘Working closely with advisory groups and learning from them; feeding back […] in an iterative fashion to policy actors/professionals as part of the research process’

These are progressive concepts and operations of engagement, in which socially-relevant knowledge is both produced with and communicated among different groups of social actors.[42] However, we believe these views were influenced by researchers’ discipline; although responses of this nature were received from across the university, 11 of the 19 (Table 1) that fell into this category came from the social sciences.

In these comments, the process of dialogue has a value in itself, allowing researchers to be open about their values and purposes when developing their investigations[16] and to demonstrate transparency, accountability and public value.[43] We note, however, that while listening, sharing and exchanging are important components of dialogue for this group, in practice the label of ‘dialogue’ was applied to a range of very different practices and strategies, not all of which are distinctively different from one-way communication or significantly reduce the sender’s role in controlling communication.[23]

### Why undertake public engagement with research?

Researchers were asked to state up to three reasons why they took part in public engagement with research. We received 246 responses to this question; in Table 3, percentages have therefore been calculated against n = 246. Sixty-nine per cent (n = 118) of the 171 respondents provided at least one reason for participation; 28 more (Table 2, n = 90) than described an activity in which they had taken part.

Researchers gave a range of reasons for engagement, including education and communication, collaboration and dialogue and to improve the quality of research. The biggest group of responses (Table 3: 15%, n = 37) focussed on the idea that public engagement enabled researchers to influence policy or policy-makers, or drive social change:

‘To effect social change’

‘To have an impact on public policy and therefore indirectly on service provision’

‘Policy may change in favour of the marginalised’

This is borne out by responses to question 25a of the CROS survey (see Method; note that. this question was only asked in the CROS survey). Thirty-two of the 57 respondents claimed they had engaged with policy-makers or end-users of research. However, there were some nuances in participation: two-thirds of researchers funded by EU frameworks said they had not undertaken any public engagement activities but would like to. This group was also the most likely to say they had engaged with policy-makers, indicating they are drawing a distinction between the ‘general’ public and the specific public of policy-makers. This disconnection may also be a result of the conservative operation and conceptualisation of engagement we described earlier; if researchers conceptualise public engagement with research as dissemination, they might not regard non-dissemination-based activities (such as discussions with policy-makers) as engagement.

The next largest group of comments (12%, n = 29) covered the idea that engaging with non-academic communities was a way to increase the quality of research by supporting more accurate research, increasing the relevance of research and relating it to the real world:

‘To keep research useful To keep research practical To potentially make a real difference’

‘Purely academic perspectives are often narrow or miss aspects which are apparent to professions in the field, or those individuals who experience it. Engaging these perspectives provides a more accurate picture.’

Earlier research uncovered reasons for undertaking public engagement which include recognising a duty to communicate research and its social and ethical implications;[44, 45] desire to further a career;[46] and the belief that engagement may lead to research that is more ‘socially, economically and environmentally viable’.[47] (p.1) All these reasons were mentioned by OU researchers, albeit in relatively small numbers (see Table 3).

Beyond these instrumental reasons, previous research has identified other factors as significant in affecting researchers’ willingness to participate in public engagement with research that are relevant to the action phases of our Catalyst for PER project. These include researchers’ previous experience and personal perceptions of their capability,[46] their urge to do what their colleagues are doing[24] and having had some training in public engagement.[45] Although we did not find evidence of all these factors in our survey (see Table 3), nevertheless, responses to question 19 in the CROS survey (n = 57) are relevant.^2^ Thirteen of the respondents had had some training in public engagement with research, whilst more than half (n = 33) had no experience of relevant training, but would like to have the opportunity. Those most likely to have had training were mid-career researchers (n = 4), with the highest demand for training coming from experienced researchers (n = 14).

### Assessing the quality of public engagement with research

Of the 171 researchers surveyed approximately half (53%, n = 92) gave no response to the question about what they saw as a successful activity and what criteria they used to make this assessment (Table 4). Thirty-nine responses were variants of ‘none’ or ‘no criteria’. Of the 40 substantial responses, 14 respondents provided more than one suggestion (n = 54).

Where criteria were offered, 18 measured success by post-event follow-up (such as emails from participants, or media interest) and 15 judged it by the behaviour of participants at the event. Only five responses mentioned some form of formal or semi-formal evaluation.

These findings, which are supported by previous research[31] and calls for routine assessments of engagement,[48] suggest that one of the key areas for development for the higher education sector is to extend the focus on developing and delivering activities to include measures of quality; in effect, to nurture a culture in which reflective practice in engaged research is valued, supported and rewarded. For example, the RCUK’s Concordat for Engaging the Public with Research states that, ‘Research organisations should themselves monitor and evaluate the efficacy of the public engagement they support…’ [48]. However, we acknowledge there can be challenges to this approach. Anecdotally, we have encountered researchers who value the idea of engagement as something undertaken voluntarily and out-of-hours, because it allows them to retain control over their activity, which could be undermined if explicit formal evaluation were demanded.[49]

### Who are the publics of public engagement with research?

The final group of institution-specific questions asked researchers for their views about the communities with which they engaged. Researchers gave a wide range of responses in their answers, including media professionals, teachers, parents, school pupils, learned societies, health-care professionals, patients, policy-makers, non-governmental organisations, companies, industrial partners, charities, community groups, voluntary and third sector organisations, university students and anonymous audiences such as viewers, listeners and readers of mass media. This diversity highlights the challenge of identifying the publics in engaged research, and the need for resources to support the processes of public formation.[50] In all, 70% (n = 121) named at least one public and 41% (n = 70) named more than one; only 1% (n = 2) said they had not engaged with any public. Twenty-nine per cent (n = 50) left the response blank.

This finding is reinforced by the researchers’ responses concerning groups with which they would *not* wish to engage. Half (55%, n = 95) left the response blank, while by far the largest single response (29%, n = 50) was some version of ‘none’, indicating researchers’ general willingness to work with a range of audiences. However, this is not quite borne out by responses to the question of which groups they *would like* to engage with, where responses were very often the same as responses to the question of which groups they *had* engaged with. Twenty-one per cent (n = 25) simply said ‘the same’ (or equivalent) and 60% (n = 70) named at least one of the same communities.

There may be many reasons for this. First, respondents may be unaware of the existence of different communities; as we noted earlier, terms that have currency in the field of public engagement research may not be readily exchangeable in other fields. For researchers in different fields, the term ‘public’ is constructed in many different ways, to the extent that language use has shifted from one ‘public’ to multiple ‘publics’ that form, re-form and overlap, depending on their interests, backgrounds, experiences and preoccupations. A restricted view of available publics may also be an artefact of researchers’ views of what constitutes public engagement; if engagement is construed as one-way transmission, this restricts audiences to those who are able and/or wish to be receivers.[31] Second, there may be restrictions on the kinds of audiences with whom researchers are able to interact; for example, funders may introduce restrictions that limit researchers to interacting with certain groups (e.g. only supporting working with industrial partners) or researchers may be working with very defined groups (e.g. specific patient groups).

Overall, these results indicate another key challenge: to explore and better understand the nature of ‘the public’ in public engagement with research and to develop interventions that support the development of meaningful research collaborations.[50]

## Conclusions: Enacting Engaged Research

This study is part of a wider action research project with the overarching aim of embedding public engagement with research into the Open University’s research culture. We have used the findings from this study, in combination with data from a second strand of research using semi-structured interviews (not reported in this paper), to shape and inform a series of interventions designed to achieve this aim. It follows that the findings, which have been discussed with senior executives at the institution,[51] our Advisory Panel,[52] the other Catalyst for PER projects, and commented on by our funders,[53] have been instrumental in identifying priority areas for intervention. Furthermore, they have been used to support professional development opportunities for researchers at all levels from postgraduate researchers to professors.[54] Here we describe three key interventions: defining engaged research, assessing and showcasing excellence, and creating a culture of reflective practice through evaluation and the development of learning resources.

### Co-producing a definition of engaged research

In our discussion of researchers’ definitions of public engagement with research, we noted some of the confusion between dissemination, dialogue and collaboration. We also offered evidence of the wide range and types of activities and publics that researchers considered to be engagement, noting the diversity both in approaches and publics. Our solution was to collaboratively produce, with researchers and Senior Executives, a definition of engaged research that could shape and inform future strategy and practice, based on evidence of the various ways that researchers and research teams from across a wide range of academic disciplines are already interacting with various kinds of ‘public’:

Engaged research encompasses the different ways that researchers meaningfully interact with various stakeholders over any or all stages of a research process, from issue formulation, the production or co-creation of new knowledge, to knowledge evaluation and dissemination.

This definition was discussed, revised and approved by the Open University’s Research Committee in July 2014[55] and subsequently approved by Senate in November 2014). It is an intervention designed to catalyse change. It has been developed in discussion with academics from across the institution, with the intention that it can be applied across all academic domains. It extends respondents’ characterisation of public engagement as predominantly a communication activity and something that is presented after the research has been completed. It addresses the challenges we discussed in the Introduction: first, the practical challenges—the who, when, how and why of engagement; second, the valuing of stakeholders’ expertise; third, the development of identity within the research process; fourth, the ownership of knowledge; and fifth, the interaction between researchers and stakeholders. (In this definition, stakeholders may include user communities, and members of the public or groups who come into existence or develop an identity in relationship to the research process.)

Co-creating this definition with academics, whilst also being informed by research, has enabled us to begin to clarify the rationales and opportunities for broadening and deepening future engagement and in particular to address the confusion about the different ways that the ‘impact agenda’[27] of innovation, enterprise, knowledge transfer and knowledge exchange connects with public engagement with research. We argue that this concept of engaged research is useful because it provides a basis for exploring the mechanisms by which a wide variety of economic and academic benefits, as well as social benefits, can be produced throughout the research process. It shifts the focus from assessing the benefits that flow from completed academic research to considering how the boundaries of academic research practice can become more permeable to participation and partnership working by people and agencies that have not traditionally been considered part of the research community. This participation can be linked to achieving various kinds of impact over time but can also usefully be considered as providing value in its own right, since the methodologies that are used to generate impact can be assessed whether or not impacts are ultimately achieved.

### Assessing quality and showcasing excellence in engaged research

Gaining institutional approval for this research-informed definition has also allowed us to explore the nature and purposes of engaged research as they play out in different academic disciplines. In turn, this has given us licence to introduce other measures, including assessments of quality. Through our work we have encountered lingering echoes of the view that being involved in engagement might be bad for a career and cause researchers, or their work, to be taken less seriously by colleagues.[45] Whilst this perception of public engagement as an activity for those less fitted to an academic career was repudiated by, for example, Bentley and Kyvik,[56] who conducted a meta-analysis of scientists’ activities in 13 countries, showing that researchers who participated in public engagement published, on average, significantly more academic publications than those who did not, we argue that there is still a job to be done to recognise and reward excellence in engaged research.

At a strategic level, we have used evidence from the research we report here to inform contributions to an institutional review of promotion criteria; operationally, we have introduced an Engaging Research Awards Scheme. Conceptualising this Awards Scheme in the light of the considerable diversity we encountered throughout our research and as we supported researchers in their engagement planning called for clear definition of the types of activities and publics that the scheme would assess. We therefore begin the process of collaboratively producing the definition of engaged research (above). Having addressed the issue of definition, we were able to develop a set of criteria for assessing the submissions, further clarifying what counts as excellence in engaged research. The criteria can be found at http://www.open.ac.uk/blogs/per/wp-content/uploads/2014/03/Engaging-Research-Awards-Scheme_assessment_protocol_2014.pdf. The winners of the inaugural scheme are listed on http://www.open.ac.uk/research/main/news/ou-announces-winners-its-first-engaging-research-awards-scheme.

### Co-creating a culture of reflective practice

Our approach to embedding engaged research within the culture of our institution has been to combine research with interventions, working across different academic disciplines and with researchers at all levels. Such an approach has strengths but also limitations, not least the degree to which the research findings and interventions can be directly linked. One can and should inform the other, but those running action research projects also have to take into account changing circumstances that are outside their control. In our case, as a small multi-disciplinary project, we have also had to judge when our interventions could be useful, and when to let our colleagues work with their stakeholders to progress their agenda.

It follows that, ultimately, for engagement and an ‘open research’ approach to become genuinely embedded within the research culture of a large and diffuse organisational structure such as a university, researchers and their publics must come to value the processes of engaged research and create a culture of reflective practice and mutual respect as partnerships develop and shift over time. This demands a combination of generic resources and bespoke support, only some of which can be provided centrally from an action research project such as ours. To address these requirements, we worked with researchers throughout the research cycle, from developing grant applications, to managing engagement during projects, and finally to considering engagement that is sustainable beyond the projects’ lifetimes. We are also developing open access resources as part of a learning programme, including a set that considers the issues and challenges facing researchers in developing digital engagement practices.

Through our research and interventions we have found that very few researchers undertake formal evaluation of engaged research, which may be a reflection of the view that engagement activities are regarded as informal and personal and therefore either less amenable to evaluation or not needing to be evaluated.[31] Thus, most researchers assessed the success of their activities in terms of short-term, informal measures of success, such as receiving positive feedback at the event. However, researchers do offer reasons for undertaking engagement that—if they are to be valued—require effective evaluation. For example, enthusing future generations about science/the arts/etc. was offered as a reason for engagement. However, without long-term evaluation it will be difficult to know whether such enthusing is successful.

We have responded to the data on measuring the quality of engaged research, supporting a seed funding scheme for active researchers wanting to explore the generation and systematic collection of evidence of the impacts from engaged research, demonstrating effects, changes and/or mutual benefits to those participating. Three awards were made under this scheme; the projects will be completed by spring 2015.

### Final thoughts

Scientists have widely ‘acknowledged the benefits *to scientists* [emphasis in original] of communicating their work with the public’,[57] (p.194) although data for researchers in other fields is scarcer. There is longitudinal evidence to support the view that the majority of scientists have a positive attitude to participating in engagement activities[44, 45, 46] but we have only partial and indirect knowledge of why publics engage.[58] Furthermore, for many UK academics the concept of public engagement with research has become intricately entangled with instrumental demands for economic and social impact.[27] The challenge for the higher education sector is to explore whether an action research-informed approach, such as the one we have outlined in this paper, can produce a more progressive agenda for engaged research, one that is meaningful and relevant for both researchers and publics.
